# High-throughput sequencing reveals differences in microbial community structure and diversity in the conjunctival tissue of healthy and type 2 diabetic mice

**DOI:** 10.1186/s12866-024-03247-y

**Published:** 2024-03-16

**Authors:** Fengjiao Li, Shuo Yang, Ji Ma, Xiaowen Zhao, Meng Chen, Ye Wang

**Affiliations:** 1 Department of Opthalmology, Shandong Provincial Hospital Affiliated to Shandong First Medical University, Jinan, 250021 Shandong China; 2 Eye Center, Second Affiliated Hospital, School of Medicine, Zhejiang University, Hangzhou, 310009 Zhejiang China; 3 Core Laboratory, The Affiliated Qingdao Central Hospital of Qingdao University, Qingdao, 266042 Shandong China; 4 Department of Opthalmology, Qingdao municipal hospital, Qingdao Municipal Hospital, No. 5 Donghai Middle Road, Shinan District, Qingdao, 266000 China

**Keywords:** High-throughput sequencing, Diabetes mellitus, Conjunctival tissue, Microbial community, Diversity

## Abstract

**Background:**

To investigate the differences in bacterial and fungal community structure and diversity in conjunctival tissue of healthy and diabetic mice.

**Methods:**

RNA-seq assays and high-throughput sequencing of bacterial 16 S rDNA and fungal internal transcribed spacer (ITS) gene sequences were used to identify differentially expressed host genes and fungal composition profiles in conjunctival tissues of diabetic BKS-db/db mice and BKS (control) mice. Functional enrichment analysis of differentially expressed genes and the correlation between the relative abundance of bacterial and fungal taxa in the intestinal mucosa were also performed.

**Results:**

Totally, 449 differential up-regulated genes and 1,006 down-regulated genes were identified in the conjunctival tissues of diabetic mice. The differentially expressed genes were mainly enriched in metabolism-related functions and pathways. A decrease in conjunctival bacterial species diversity and abundance in diabetic mice compared to control mice. In contrast, fungal species richness and diversity were not affected by diabetes. The microbial colonies were mainly associated with cellular process pathways regulating carbohydrate and lipid metabolism, as well as cell growth and death. Additionally, some interactions between bacteria and fungi at different taxonomic levels were also observed.

**Conclusion:**

The present study revealed significant differences in the abundance and composition of bacterial and fungal communities in the conjunctival tissue of diabetic mice compared to control mice. The study also highlighted interactions between bacteria and fungi at different taxonomic levels. These findings may have implications for the diagnosis and treatment of diabetes.

## Introduction

Type 2 diabetes mellitus (T2DM) is the most common type of diabetes and is characterized by insulin resistance, insulin deficiency, or β-cell failure [1, 2]. The onset and progression of T2DM are mainly related to factors such as obesity, family history, and diet, and its prevalence is increasing globally [3, 4]. Concurrently, dry eye and other diseases of the ocular surface caused by diabetes are becoming increasingly prevalent in clinical practice [5]. Ocular surface diseases refer to disorders that impair the normal structure and function of the ocular surface, such as the cornea and conjunctiva [6]. Epidemiological data suggest that the rising number of diabetes cases has led to an increase in the number and severity of ocular surface diseases. As patients age, diabetes is gradually becoming one of the most common risk factors for ocular diseases [7]. Diabetes mellitus is characterized by hyperglycemia, and numerous studies have shown that it makes patients more susceptible to infectious symptoms throughout the body, including ocular infections of the eyelids, conjunctiva, and cornea [8]. This further leads to complications after ocular surgery [9] and a poorer visual prognosis [10]. The increase in glucose levels in the skin, urine, mucosa, and tears disrupts the original balance of this tissue and provides favorable conditions for the growth of microorganisms in this area [11]. Conjunctival lesions have been reported in 86% of diabetic patients, with varying symptoms and severity [12]. The conjunctival flora begins to develop shortly after birth, and its composition depends on age, season, environmental conditions, immune status, and general hygiene. Some members of the putrefying conjunctival flora act pathogenically when immune function is impaired, which can lead to serious infections [13].

Under normal circumstances, the ocular surface is constantly exposed to the external environment and is a key component of the body’s defense system [14, 15]. Dysfunctions of the ocular surface ecosystem have been linked to various ocular infections. Common bacterial isolates associated with ocular infections include Staphylococcus aureus [16], coagulase-negative staphylococci [17], Streptococcus pneumoniae [18], and Pseudomonas aeruginosa [19]. Previous studies mainly relied on bacterial culture methods for strain identification. However, these methods have limitations as they can only identify specific bacterial species, and various factors such as the in vitro environment and incubation time during bacterial culture can impact bacterial growth [20, 21]. The advent of next-generation sequencing has enabled better characterization of microbial communities through DNA-based assays (i.e., microbiome), overcoming the limitations of traditional culture techniques [22]. Several studies have identified a low diversity of microbial genera on the ocular surface, further supporting this notion [23, 24].

Although many studies focus on analyzing the abundance and diversity of bacteria in the eye, it should be noted that bacteria are not the only microorganisms that colonize the ocular surface. Fungi and viruses also contribute to the ocular surface microbiome, with bacterial reads accounting for approximately 98% of identified microbiome sequences and the remaining 2% consisting of fungi and viruses [25, 26]. Among the fungi, a core microbiome of four genera has been identified, including Aspergillus, Coccus, Malassezia, and Erythrobacter Shiba and Simidu [27]. Previous studies using culture methods have reported fungal positivity rates in normal conjunctival sacs ranging from 3 to 28%, with up to 87% of conjunctival sac swabs failing to culture any microorganisms [28, 29]. With the development of second-generation high-throughput sequencing tools, it is now possible to directly sequence and classify samples without culture. The RNA-seq, 16 S rDNA, and ITS techniques used in this study provide a more comprehensive understanding of the ocular surface bacterial and fungal community structure and species diversity. By analyzing the transcriptome differences and bacterial and fungal community results of conjunctival tissues from healthy and diabetic mice, this study contributes to our knowledge of the ocular surface microbiome and may shed light on bacterial-fungal interactions.

## Methods

### Study subjects and housing

12 male BKS and BKS-db/db mice, aged six to eight weeks, were obtained from the Model Animal Research Center of Nanjing University. All mice were housed in a specific pathogen-free environment, with a 12-hour light and 12-hour dark cycle, at a temperature of 22 ± 2℃ and a humidity of 50 ± 5%. Mice were provided with sufficient food and water and free space to move around during the incubation period. The mice were allowed to acclimate for one week before the start of the experiments. All animal experimental protocols were conducted and approved in accordance with the guidelines established by the Institutional Animal Care and Use Committee, Qingdao University (Qingdao, Shandong, China). All procedures involving the animals adhered to the guidelines outlined by the Association for Research and the Vision and Ophthalmology statement regarding the use of animals in ophthalmic and vision research. The study is reported in accordance with Animal Research: Reporting of In Vivo Experiments guidelines (https://arriveguidelines.org).

#### Conjunctival tissue Sample Collection

Both eyes of each mouse were screened and sampled for sequencing. The entire extracted tissue containing the orbital conjunctiva was removed and frozen for subsequent RNA and DNA extraction.

### RNA extraction and sequencing

Total RNA was extracted from the tissues using Trizol reagent (Invitrogen, Carlsbad, CA, USA), and RNA quality was assessed using RNase-free agarose gel electrophoresis with the Agilent 2100 Bioanalyzer (Agilent Technologies, Palo Alto, CA, USA). After extraction, eukaryotic mRNA was enriched with oligo (dT) beads. The enriched mRNA was then fragmented using a fragmentation buffer and reverse transcribed into short fragments using the NEBNext Ultra RNA Library Prep Kit for Illumina (NEB #7530, New England Biolabs, Ipswich, MA, USA). The purified double-stranded cDNA fragments were end-repaired, had an A base added, and were ligated to Illumina sequencing adapters. The ligation reaction was purified with AMPure XP Beads (1.0X). Bound fragments were size-selected by polymerase chain reaction (PCR) amplification. The resulting cDNA library was sequenced using Illumina Novaseq6000 software (Gene Denovo Biotechnology Co., Ltd., Guangzhou, China).

### Differential gene expression analysis

The DEseq2 package in R v4.0.4 was utilized to perform differential expression analysis between normal and diabetic conjunctival tissue samples. The criteria for screening differentially expressed genes (DEGs) were set at false discovery rate (FDR) < 0.05 and log2|Fold Change|≥1.

### Enrichment analysis of gene functions and pathways

Gene Ontology (GO) is an internationally standardized system for gene function classification that defines rigorously controlled concepts and a dynamically updated vocabulary to comprehensively characterize genes and their products in any organism. GO consists of three ontologies, namely Molecular Function, Cellular Component, and Biological Processes, which map all DEGs to GO terms in the Gene Ontology database (http://www.geneontology.org/), count the number of genes in each term, and determine significantly enriched GO terms by hypergeometric tests compared to the genomic background. The Kyoto Encyclopedia of Genes and Genomes (KEGG) is the main public pathway-related database, which identifies significantly enriched metabolic or signal transduction pathways in DEGs compared to the genome-wide background. Reactome is a free online database of biological pathways, with the reaction as the core unit of its data model. Entities involved in a reaction, such as nucleic acids, proteins, complexes, and small molecules, form a network of biological interactions and are grouped into pathways, which include signal transduction, innate and acquired immune function, transcriptional regulation, translation, apoptosis, and classically mediated metabolism. Enrichment analysis of gene functions and pathways was considered statistically significant if the FDR value was less than 0.05.

### Protein-protein interaction network construction

Differential analysis of genes was conducted, and protein-protein interaction (PPI) networks were constructed using the online analysis tool, Metascape (https://metascape.org/gp/index.html#/main/step1). The PPI networks were further screened for hub genes using MCODE in Cytoscape (v3.8.2) software.

### 16 S rDNA and ITS extraction and sequencing

Microbial DNA was extracted using the HiPure Soil DNA Kits (Magen, Guangzhou, China) following the manufacturer’s instructions. For amplification of the V3-V4 region of the ribosomal RNA gene, primers 341F: CCTACGGGNGGCWGCAG and 806R: GGACTACHVGGGTATCTAAT were used for PCR. The eukaryotic ribosomal RNA gene was amplified using the ITS3_KYO2F 5’-GATGAAGAACGYAGYRAA-3’ and ITS4R 5’-TCCTCCGCTTATTGATATGC-3’ primers. Amplification conditions were as follows: 95 °C for 2 min, followed by 27 cycles of 98 °C for 10 s, 62 °C for 30 s, and 68 °C for 30 s, with a final extension at 68 °C for 10 min. Amplicons were extracted from 2% agarose gels and purified using the AxyPrep DNA Gel Extraction Kit (Axygen Biosciences, Union City, CA, USA). The ABI StepOnePlus Real-Time PCR System (Life Technologies, Foster City, USA) was used to quantify the purified amplicons, which were then aggregated at equimolar levels according to standard protocols. The Illumina platform was used to end-sequence 2,250 amplicons. Raw read data were deposited in the NCBI Sequence Read Archive database.

### OTU clustering and species annotation

The sequences were clustered into operational taxonomic units (OTUs) at 97% similarity using Uparse software. Intergroup Venn analysis was performed in R software to identify unique and universal OTUs. Representative sequences were classified using a plain Bayesian model with a confidence threshold range of 0.8-1 compared to the unit database.

### Diversity analysis

In QIIME2, the alpha diversity indices including ACE, Chao1, Simpson, Shannon, Pielou, Sobs, goods_coverage, and PD were calculated. Sparsity curves and rank abundance curves of OTU were plotted in QIIME2. Alpha indices were compared between groups using Welch’s *t*-test, Wilcoxon rank test, Tukey’s HSD test, and Kruskal-Wallis H test in the R project. To calculate beta diversity, weighted and unweighted UniFrac distance matrices were generated using QIIME2, and multivariate statistical techniques were used in R software to calculate weighted and unweighted UniFrac distances. Statistical analysis was performed using the Welch t-test, Wilcoxon rank test, Tukey’s HSD test, Kruskal-Wallis H test, Adonis (also known as Permanova) test, and Anosim test in R software.

### Functional prediction

Tax4Fun (version 1.0) was used to analyze the KEGG pathway of OTUs, while FUNGuild (version 1.0) was used to analyze functional groups of fungi for OTUs. Functional differences between groups were analyzed using the Welch t-test, Wilcoxon rank test, Kruskal-Wallis H test, and Tukey’s HSD test with the vegan package in R software.

### Statistical analysis and mapping

GraphPad Prism 8 software was used to perform the calculations and plotting the images. The comparisons between the two groups were made using the *t*-test. Correlation analysis was performed using the Pearson R test. Statistical significance was set at *p* < 0.05.

## Results

### Transcriptome analysis of samples

**Fig. 1:**
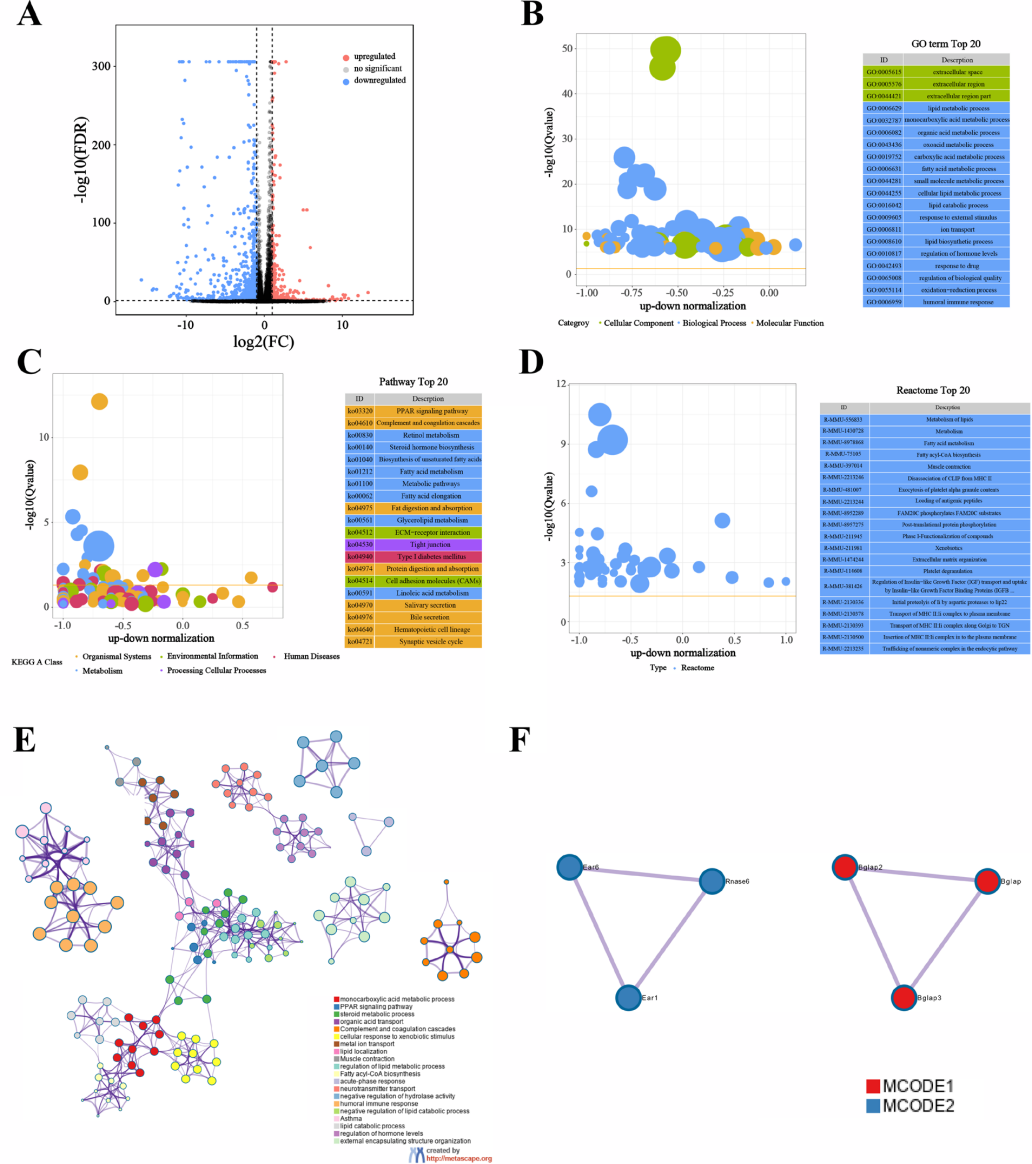
Differential expression and functional analysis of genes in control and diabetic groups. (A) Volcano plot showing the differentially expressed genes in control (WT-TC) and diabetic (DB-TC) groups. 449 genes were upregulated (red) and 1,006 genes were downregulated (blue). The x axis is log2 (Fold Change), which is the multiple of the difference between two groups of samples and the y axis showed the log P-value which calculated by t-test. The cut-off is 1.3=-log10 (0.05). (B) Gene Ontology (GO) functional enrichment analysis of differentially expressed genes. The top enriched GO terms were related to metabolism, such as the lipid metabolic process and the monocarboxylic acid metabolic process. (C) Kyoto Encyclopedia of Genes and Genomes (KEGG) pathway enrichment analysis of differentially expressed genes. The top enriched pathways were metabolic pathways, such as the PPAR signaling pathway and retinol metabolism. (D) Reactome pathway analysis of differentially expressed genes. The top enriched pathways were related to various metabolic processes, such as the metabolism of lipids and fatty acid metabolism. (E) Protein-protein interaction (PPI) network analysis of differentially expressed genes using Metascape. The network was visualized with gene information, and nodes were colored based on their degree of connectivity. (F) The largest subnetworks in the PPI network were selected and visualized using the MCODE plug-in in Cytoscape. MCODE1 functions were mainly enriched in the metabolism of lipids and the response to vitamins

In this study, we collected three tissue samples each from the control and diabetic groups, referred to as WT-TC and DB-TC, respectively. After generating the gene expression matrix, we identified 449 upregulated and 1,006 downregulated differentially expressed genes (DEGs) (Fig. 1A). Functional enrichment analysis of the DEGs showed that they were mainly associated with metabolism-related Gene Ontology (GO) terms, such as lipid and monocarboxylic acid metabolic processes (Fig. 1B). KEGG pathway analysis revealed that the DEGs were enriched in metabolic pathways such as the PPAR signaling pathway and retinol metabolism (Fig. 1C). Moreover, Reactome pathway analysis demonstrated that the DEGs were also enriched in various metabolic pathways, including Metabolism of lipids and Fatty acid metabolism (Fig. 1D). To obtain protein-protein interaction (PPI) networks, the DEGs were analyzed using the Metascape website, and the resulting networks were visualized (Fig. 1E). The MCODE plug-in in Cytoscape was used to identify characteristics of each node in the network graph, and the largest subnetworks were selected and visualized (Fig. 1F). MCODE1 functions were mainly enriched in the metabolism of lipids and response to vitamins.

### 16 S rDNA sequencing results and changes in microbial diversity

**Fig. 2:**
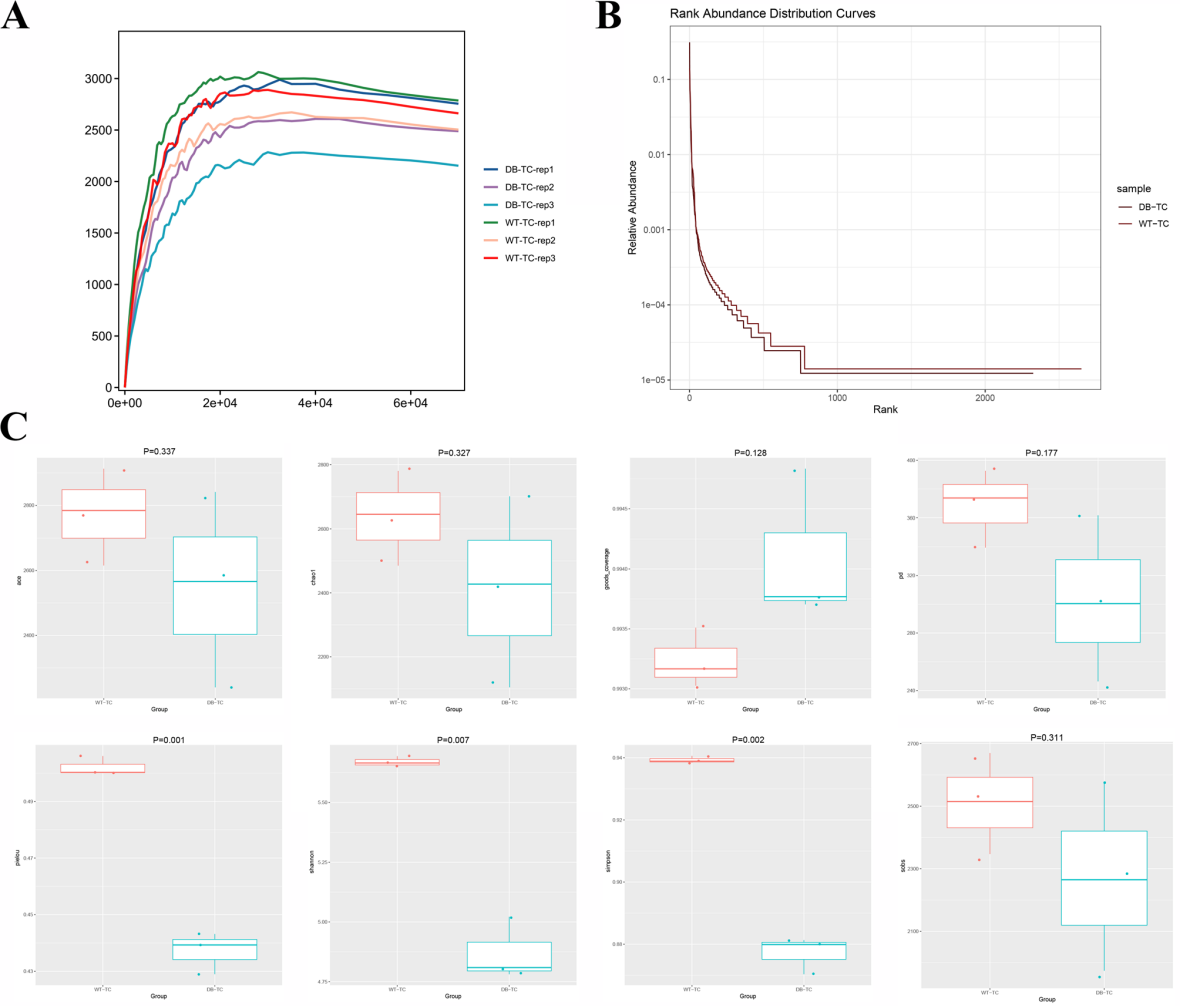
Analysis of microbial diversity in control and diabetic groups using 16 S rDNA sequencing. (A) Dilution curve showing the reasonableness of the sample size with the increase of sample size. The x axis represents the number of sequencing samples randomly selected from a certain sample, and the y axis represents the number of OTUs that can be constructed based on this number of sequencing samples to reflect the depth of sequencing. (B) Rank abundance curve showing the abundance and evenness of conjunctival flora in the WT-TC and DB-TC groups. (C) Alpha diversity indices including Pielou, Shannon, and Simpson indices, reflecting the abundance and diversity of microbial communities in the samples. The overall index of the WT-TC group was higher than that of the DB-TC group, indicating a higher degree of microbial community diversity in the control group. The occurrence of diabetes caused a decrease in species richness and diversity (*p* < 0.05). The goods_coverage index for each group was greater than 0.99, indicating high credibility of the data

To verify the reasonableness of the sample size, we examined the dilution curve and rank abundance curve. The dilution curve (Fig. 2A) showed that the results tended to become more reasonable as the sample size increased. The rank abundance curve (Fig. 2B) indicated that the conjunctival flora’s abundance was higher and more evenly distributed in the WT-TC and DB-TC groups, but the DB-TC group’s abundance was lower than that of the WT-TC group.

Alpha diversity reflects the diversity of species in a single sample, and various indices such as Pielou index, Sobs index, Chao index, and ACE index reflect the community’s abundance, while the Shannon index, PD index, and Simpson index reflect the community’s diversity. The goods_coverage index reflects the sequencing depth, and a higher value indicates higher credibility. The goods_coverage index for each group was greater than 0.99, indicating highly credible data. Alpha diversity indices (Fig. 2C) with significant differences under different conditions were screened by a rank sum test, including the Pielou, Shannon, and Simpson indices. The overall index of the WT-TC group was higher than that of the DB-TC group, indicating higher microbial community diversity in the former. Diabetes caused a decrease in species richness and diversity (*p* < 0.05).

**Fig. 3:**
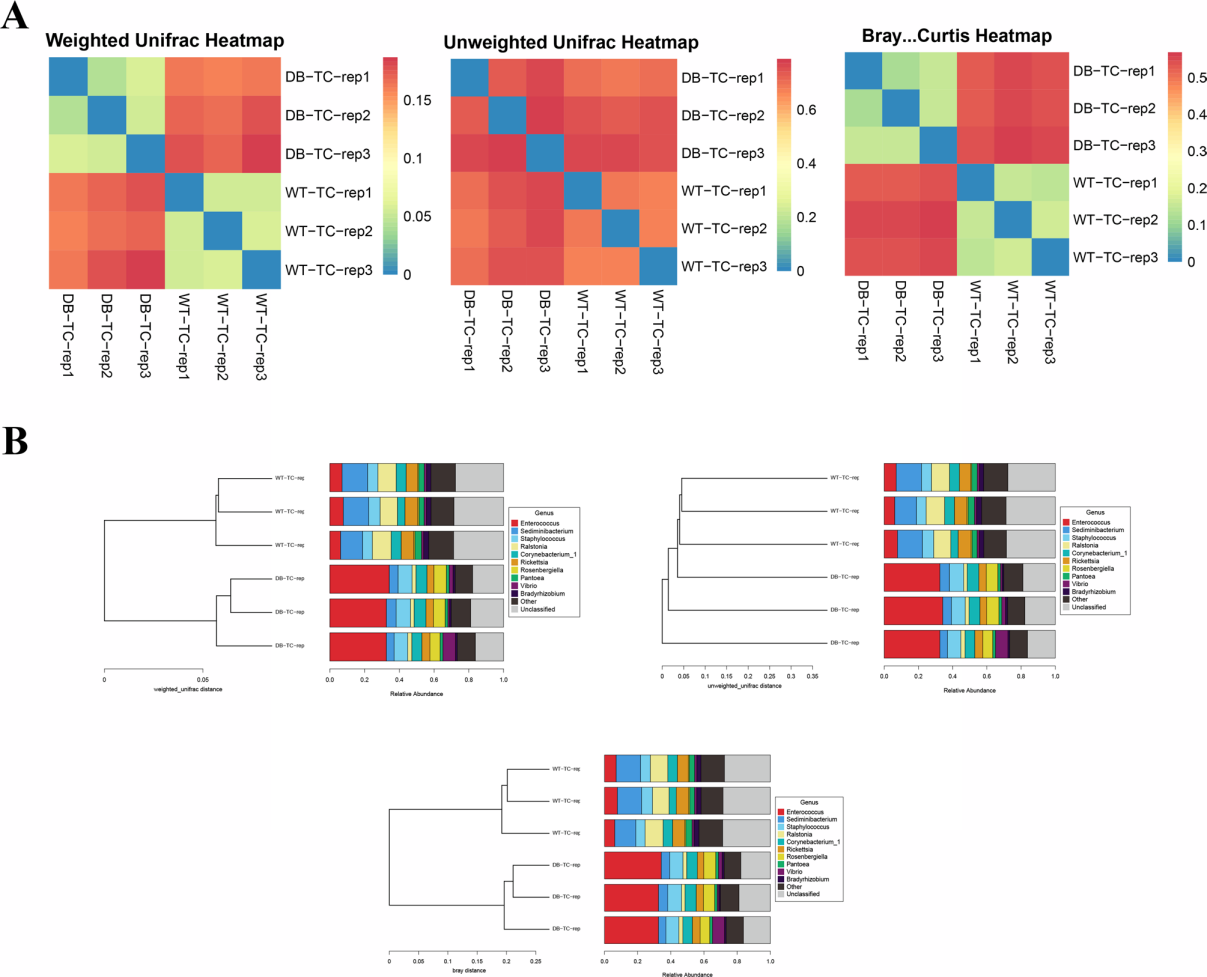
mBeta diversity was used to compare the magnitude of differences between pairs of samples in terms of species diversity. (A) The beta diversity matrix heat map obtained using Bray-Curtis, weighted UniFrac, and unweighted UniFrac analyses. Samples with similar beta diversity were clustered together, revealing the similarity between them. The beta diversity between the two groups was well-clustered and could be distinguished within a certain range. Based on the beta diversity distance matrix, the samples were classified into UPGMA classification trees, where the more similar samples had shorter common branches. (B) The results of cluster analysis for all samples, indicating that the WT-TC and DB-TC groups had similar colony results but differed in their compositional abundance

mBeta diversity was used to compare the magnitude of differences between pairs of samples in terms of species diversity. Bray-Curtis, weighted UniFrac, and unweighted UniFrac were used. Figure 3A is the beta diversity matrix heat map. The beta diversity data were visualized graphically, and the samples were clustered by clustering samples with similar beta diversity together, reflecting the similarity between samples. As seen from the figure, the respective beta diversity between the two groups was well clustered, and the groups could be clustered within a certain range. Based on the beta diversity distance matrix information, the samples were classified into UPGMA classification trees. The cluster analysis results for all samples are shown in Fig. 3B, indicating that the WT-TC and DB-TC groups have similar colony results but differ in compositional abundance.

### Elaborating the distribution of bacterial flora through 16 S rDNA sequencing

**Fig. 4:**
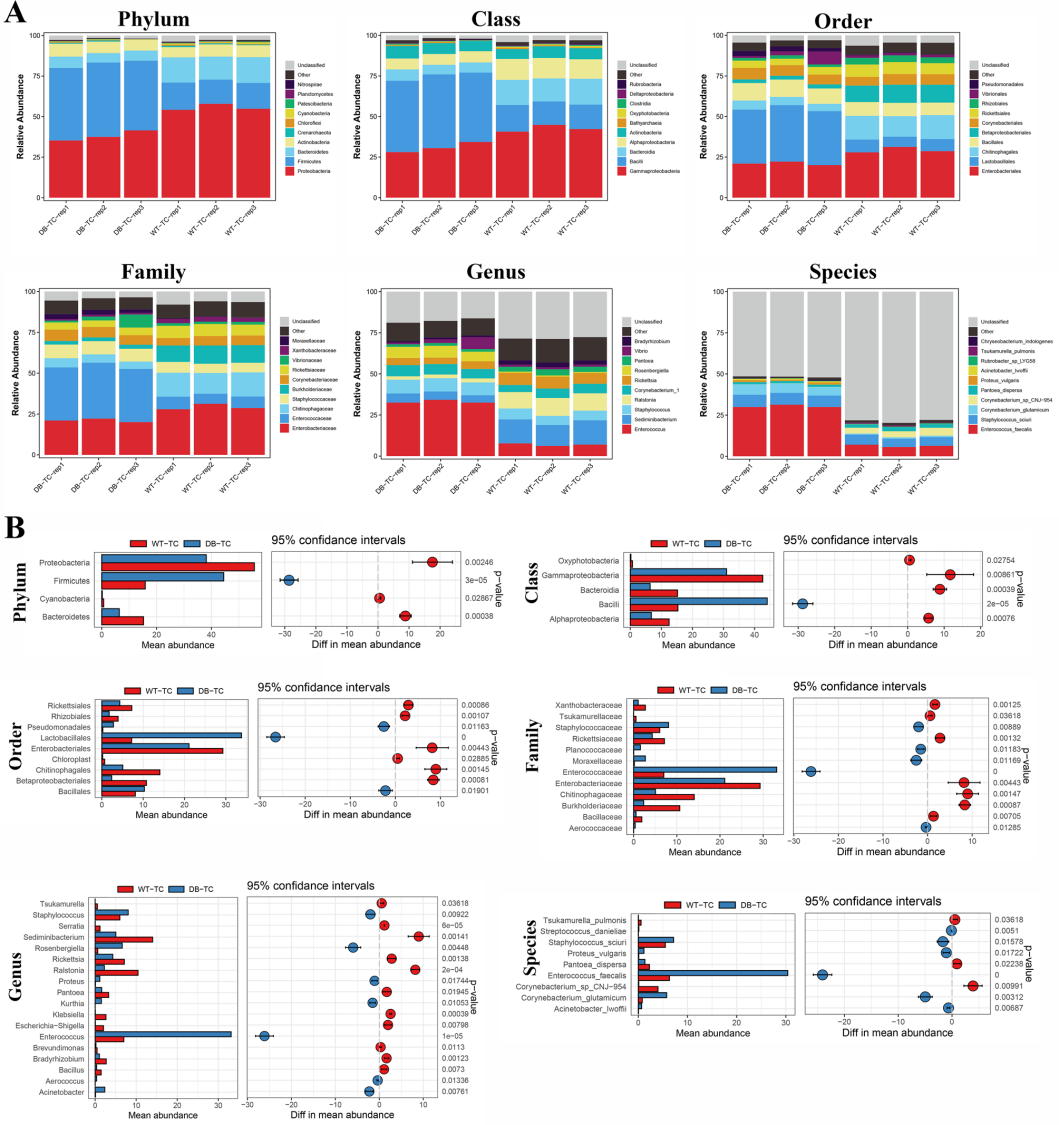
Microbial diversity in conjunctival flora of diabetic and control mice. (A) Relative abundance of the top 10 phyla, classes, orders, families, genera, and species in both groups of mice. (B) Comparison of the abundance of major phyla, classes, orders, families, genera, and species between diabetic and control mice

At the phylum level, the top 10 phyla of conjunctival flora in both groups of mice were *Proteobacteria*, *Firmicutes*, *Bacteroidetes*, *Actinobacteria*, *Crenarchaeota*, *Chloroflexi*, *Cyanobacteria*, *Patescibacteria*, *Planctomycetes*, and *Nitrospirae*. The four predominant phyla were *Proteobacteria*, *Firmicutes*, *Bacteroidetes*, and *Cyanobacteria* (Fig. 4A). Compared to the control group, the abundance of *Firmicutes* increased in the diabetic group, while the proportions of *Proteobacteria*, *Bacteroidetes*, and *Cyanobacteria* decreased significantly (*p* < 0.05) (Fig. 4B).

At the class level, the top 10 classes of conjunctival flora in both groups of mice were *Gammaproteobacteria*, *Bacilli*, *Bacteroidia*, *Alphaproteobacteria*, *Actinobacteria*, *Bathyarchaeia*, *Oxyphotobacteria*, *Patescibacteria*, *Clostridia*, and *Deltaproteobacteria*. The five dominant classes were *Gammaproteobacteria*, *Bacilli*, *Bacteroidia*, *Alphaproteobacteria*, and *Actinobacteria* (Fig. 4A). Compared to the control group, the abundance of Bacilli increased in the diabetic group, while the abundance of *Gammaproteobacteria*, *Bacteroidia*, *Alphaproteobacteria*, and *Actinobacteria* proportions were significantly reduced (*p* < 0.05) (Fig. 4B).

At the order level, the top 10 orders of conjunctival flora in both groups of mice were *Enterobacteriales*, *Lactobacillales*, *Chitinophagales*, *Bacillales*, *Betaproteobacteriales*, *Corynebacteriales*, *Rickettsiales*, *Rhizobiales*, *Vibrionales*, and *Pseudomonadales* (Fig. 4A). Compared to the control group, the abundance of *Lactobacillales*, *Pseudomonadales*, and *Bacillales* increased in the diabetic group, while the proportion of *Rickettsiales*, *Rhizobiales*, *Enterobacteriales*, *Chitinophagales*, and *Betaproteobacteriales* were significantly reduced (*p* < 0.05) (Fig. 4B).

At the Family level, the top 10 families in terms of relative abundance of conjunctival flora in both groups of mice were *Enterobacteriaceae*, *Enterococcaceae*, *Chitinophagaceae*, *Staphylococcaceae*, *Burkholderiaceae*, *Corynebacteriaceae*, *Rickettsiaceae*, *Vibrionaceae*, *Xanthobacteraceae*, and *Moraxellaceae* (Fig. 4A). Compared to the control group, the abundance of *Staphylococcaceae*, *Moraxellaceae*, and *Enterococcaceae* increased in the diabetic group, while the proportions of *Xanthobacteraceae*, *Rickettsiaceae*, *Enterobacteriaceae*, *Chitinophagaceae*, and *Burkholderiaceae* were significantly reduced (*p* < 0.05) (Fig. 4B).

At the Genus level, the top 10 genera in terms of relative abundance of conjunctival flora in both groups of mice were *Enterococcus*, *Sediminibacterium*, *Staphylococcus*, *Ralstonia*, *Corynebacterium_1*, *Rickettsia*, *Rosenbergiella*, *Pantoea*, *Vibrio*, and *Bradyrhizobium* (Fig. 4A). Compared to the control group, there was an increase in the abundance of *Staphylococcus*, *Rosenbergiella*, *Moraxellaceae*, and *Enterococcus* in the diabetic group, while the proportions of *Sediminibacterium*, *Rickettsia*, *Ralstonia*, *Pantoea*, and *Bradyrhizobium* were significantly reduced (*p* < 0.05) (Fig. 4B).

At the Species level, the top 10 species in terms of relative abundance of conjunctival flora in both groups of mice were *Enterococcus_faecalis*, *Staphylococcus_sciuri*, *Corynebacterium_glutamicum*, *Corynebacterium_sp_*CNJ-954, *Pantoea_dispersa*, *Proteus_vulgaris*, *Acinetobacter_lwoffii*, *Rubrobacter_sp*_LYG58, *Tsukamurella_pulmonis*, and *Chryseobacterium_indologenes* (Fig. 4A). Compared to the control group, the abundance of *Staphylococcus_sciuri*, *Proteus_vulgaris*, *Enterococcus_faecalis*, and *Acinetobacter_lwoffii* increased in the diabetic group, while the proportions of *Tsukamurella_pulmonis*, *Pantoea_dispersa*, and *Corynebacterium_sp*_CNJ-954 were significantly reduced (*p* < 0.05).

### ITS sequencing results and changes in microbial diversity

**Fig. 5:**
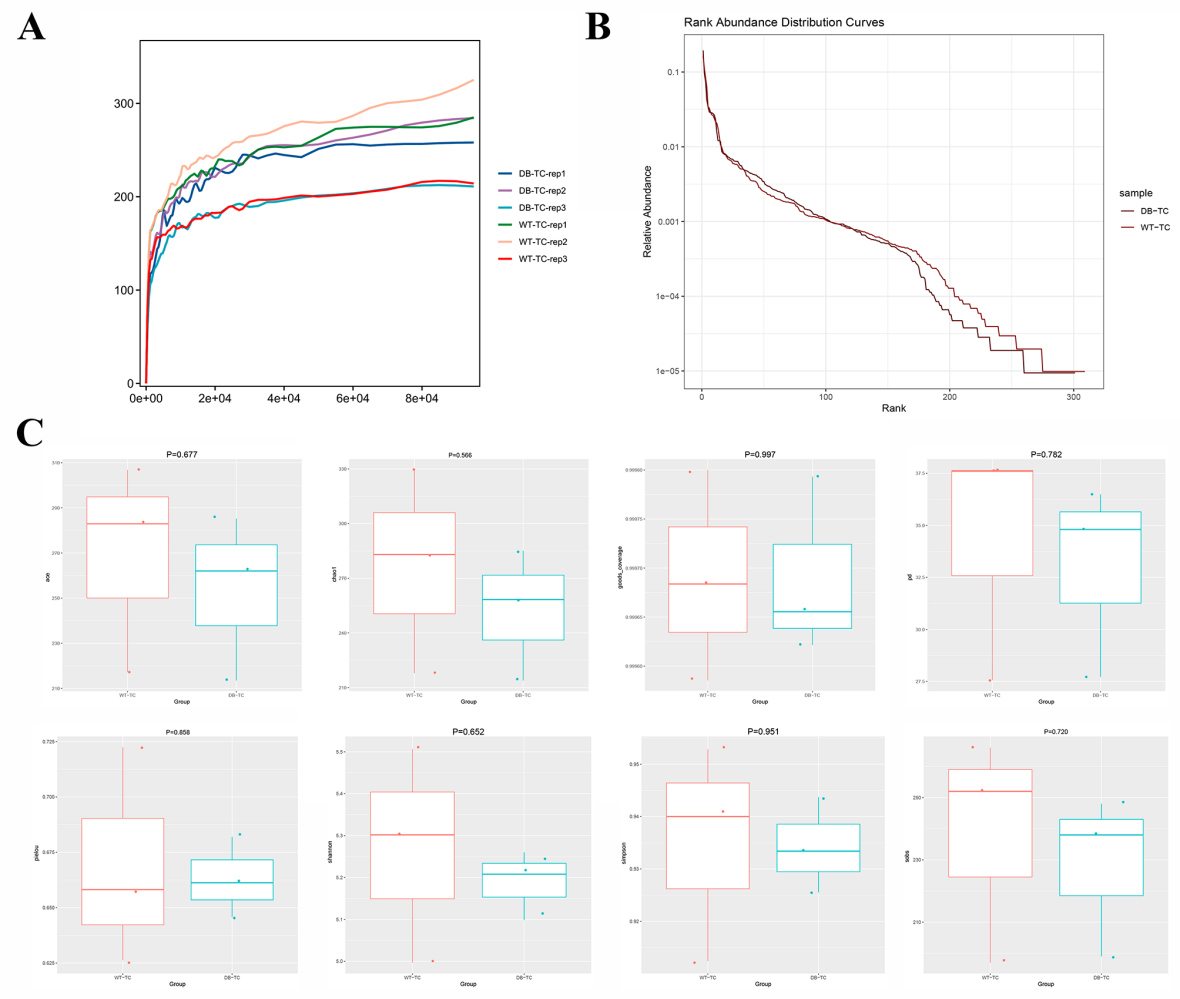
ITS sequencing results. (A) The dilution curve of conjunctival flora samples, which indicates a reasonable increase in results with increasing sample size. The x axis represents the number of sequencing samples randomly selected from a certain sample, and the y axis represents the number of OTUs that can be constructed based on this number of sequencing samples to reflect the depth of sequencing. (B) The abundance rank curve of the WT-TC and DB-TC groups, revealing that the abundance of conjunctival flora in the two groups is higher and more evenly distributed, while the abundance of the DB-TC group is lower than that of the WT-TC group. (C) Alpha diversity analysis revealed no statistical difference in fungal species abundance and diversity between the two groups (*p* > 0.05)

**Fig. 6:**
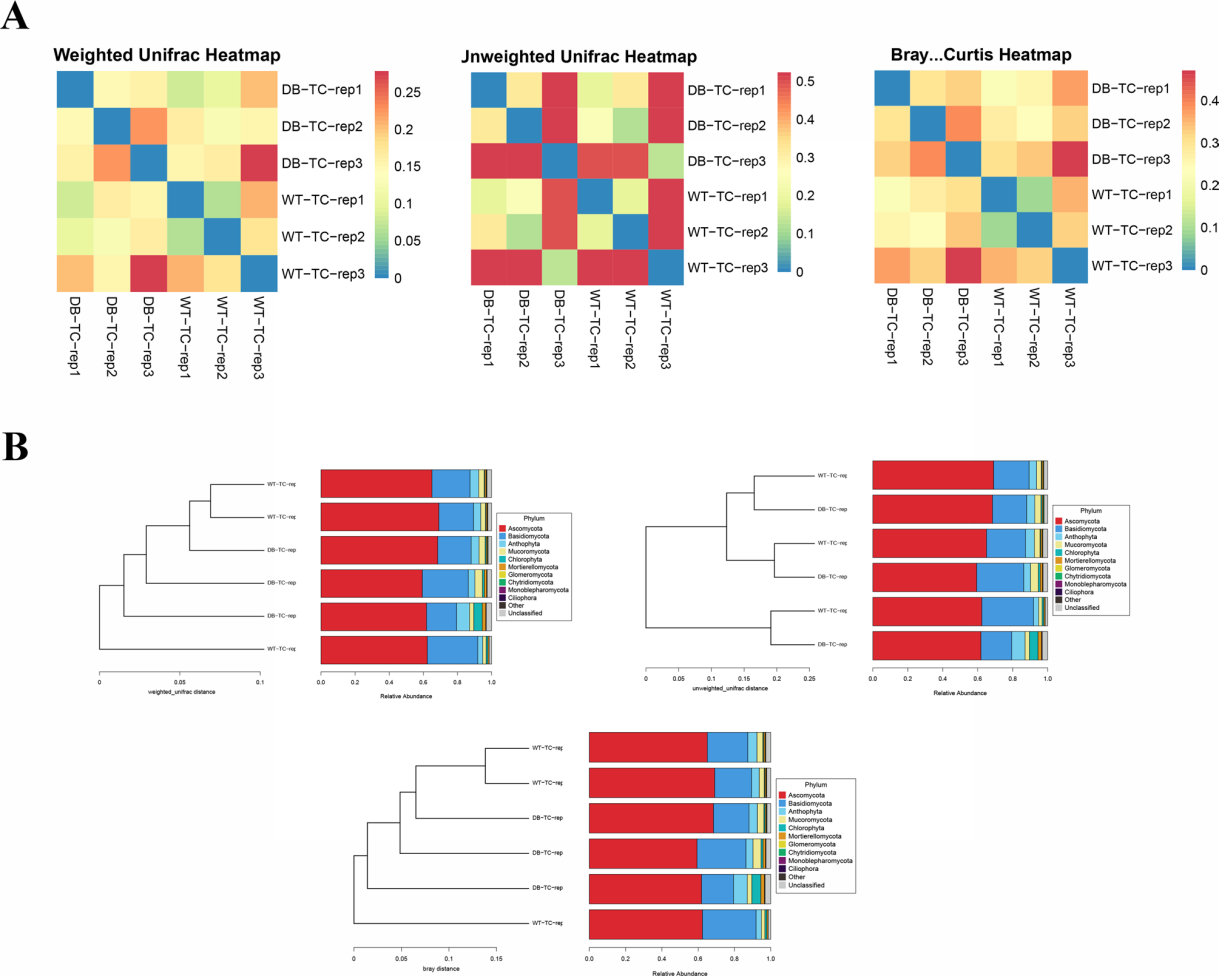
Results of Beta diversity analysis. (A) Using clustering analysis, poor clustering was shown between the two groups. (B) The fungal colony composition of the WT-TC and DB-TC groups. There are no significant differences between all groups, as indicated by the clustering analysis. These findings suggest that the occurrence of diabetes did not impact the fungal colony composition in the conjunctiva

The dilution curve (Fig. 5A) shows that the results become more reliable with increasing sample size. The abundance rank curve (Fig. 5B) suggests that conjunctival flora in both the WT-TC and DB-TC groups are more abundant and evenly distributed than in the control group. The abundance of conjunctival flora is lower in the DB-TC group than in the WT-TC group. Alpha diversity analysis did not show any statistically significant difference in fungal species abundance and diversity between the two groups (*p* > 0.05), indicating that diabetes did not significantly impact the abundance and diversity of fungal species (Fig. 5C). Beta diversity analysis showed poor beta diversity clustering between the two groups (Fig. 6A), and the clustering analysis of all samples indicated that the fungal colony composition of the WT-TC and DB-TC groups did not significantly differ (Fig. 6B).

### Elaborating the distribution of fungal mycota by ITS sequencing

Table 1 presents the results of ITS sequencing for the two groups of samples. Most of the fungi identified in the samples belonged to the *phylum Ascomycota*, class *Sordariomycetes*, order *Sordariales*, family *Bolbitiaceae*, and genus *Agrocybe*. At the species level, the highest frequency OTU1 matched four different fungal ITS regions: *Agrocybe pediades*, *Serendipita indica*, *Humicola grisea*, and *Rhizopus arrhizus*.

**Table 1:** The results of ITS sequencing for the two groups of samples. Most of the fungi identified in the samples belonged to the *phylum Ascomycota*, class *Sordariomycetes*, order *Sordariales*, family *Bolbitiaceae*, and genus *Agrocybe*. At the species level, the highest frequency OTU1 matched four different fungal ITS regions: *Agrocybe pediades*, *Serendipita indica*, *Humicola grisea*, and *Rhizopus arrhizus*

Classification	Name of fungi	Relative abundance of fungi					
		DB-TC-rep1	DB-TC-rep2	DB-TC-rep3	WT-TC-rep1	WT-TC-rep2	WT-TC-rep3
Phylum	*Ascomycota*	68.451	59.405	61.868	69.107	65.060	62.344
	*Basidiomycota*	19.567	26.950	17.617	20.330	22.339	29.596
	*Anthophyta*	4.650	3.891	7.591	4.261	5.048	2.912
	*Mucoromycota*	3.545	4.403	2.485	2.688	3.216	1.969
	*Chlorophyta*	0.657	1.104	4.957	0.369	0.423	1.006
	*Mortierellomycota*	0.643	1.375	2.051	0.634	0.783	0.758
	*Glomeromycota*	0.005	0.011	0	0.336	0.328	0
	*Chytridiomycota*	0.423	0.005	0	0.084	0.090	0
	*Monoblepharomycota*	0.001	0	0.333	0	0	0.019
	*Ciliophora*	0	0.264	0	0	0.004	0
Class	*Sordariomycetes*	27.241	27.339	24.479	25.479	28.034	33.723
	*Agaricomycetes*	17.489	23.047	15.343	18.869	20.564	25.582
	*Eurotiomycetes*	7.763	8.272	6.308	10.727	11.048	10.969
	*Eudicotyledonae*	4.650	3.891	7.591	4.261	5.048	2.912
	*Dothideomycetes*	4.371	4.488	2.016	3.763	4.145	3.584
	*Mucoromycetes*	3.545	4.403	2.485	2.688	3.216	1.969
	*Mortierellomycetes*	0.643	1.375	2.051	0.634	0.783	0.758
	*Leotiomycetes*	1.129	0.569	1.799	0.742	0.815	1.072
	*Tremellomycetes*	0.640	2.029	0.398	0.170	0.233	1.309
	*Chlorophyceae*	0	0.761	2.659	0.083	0.121	0.427
Order	*Sordariales*	15.491	15.802	11.683	13.261	14.300	19.077
	*Agaricales*	9.398	12.369	10.374	10.238	11.417	16.197
	*Hypocreales*	7.780	8.727	7.674	7.752	8.917	11.643
	*Sebacinales*	8.062	10.187	4.373	8.103	8.557	8.832
	*Eurotiales*	4.992	6.779	6.248	9.386	9.604	9.008
	*Pleosporales*	4.189	4.153	2.008	3.652	3.992	3.285
	*Mucorales*	3.545	4.403	2.485	2.688	3.216	1.969
	*Brassicales*	2.237	2.957	2.803	3.273	3.901	1.411
	*Mortierellales*	0.643	1.375	2.051	0.634	0.783	0.758
	*Asterales*	2.0884	0.787	0.023	0.745	0.890	1.300
Family	*Bolbitiaceae*	8.086	11.697	9.123	9.280	10.397	14.078
	*Chaetomiaceae*	7.948	8.628	6.776	7.261	7.803	10.945
	*Serendipitaceae*	8.062	10.187	4.373	8.103	8.557	8.832
	*Trichocomaceae*	3.114	5.684	4.136	6.414	6.940	6.840
	*Lasiosphaeriaceae*	4.088	3.702	2.359	3.696	4.146	4.074
	*Nectriaceae*	3.672	2.792	1.014	3.032	3.306	5.213
	*Rhizopodaceae*	3.545	4.307	2.480	2.543	3.073	1.713
	*Brassicaceae*	2.237	2.958	2.803	3.273	3.901	1.411
	*Stachybotryaceae*	2.248	2.695	2.167	2.045	2.419	1.517
	*Aspergillaceae*	1.879	1.094	2.111	2.972	2.664	2.168
Genus	*Agrocybe*	8.086	11.697	9.123	9.280	10.397	14.078
	*Serendipita*	8.062	10.187	4.373	8.103	8.557	8.832
	*Talaromyces*	3.114	5.684	4.136	6.414	6.940	6.840
	*Humicola*	3.562	2.798	2.217	2.792	2.928	4.074
	*Rhizopus*	3.545	4.307	2.480	2.543	3.073	1.713
	*Aspergillus*	1.193	1.043	2.106	2.369	2.021	2.029
	*Fusarium*	1.650	1.800	0.896	1.510	1.667	1.130
	*Stachybotrys*	1.267	1.117	1.369	1.043	1.247	0.994
	*Mortierella*	0.643	1.375	2.051	0.634	0.783	0.758
	*Setophoma*	0.960	0.464	0.496	1.094	1.272	0.615
Species	*Agrocybe pediades*	8.086	11.697	9.123	9.280	10.397	14.078
	*Serendipita indica*	8.062	10.187	4.373	8.103	8.557	8.832
	*Humicola grisea*	3.562	2.798	2.217	2.792	2.928	4.074
	*Rhizopus arrhizus*	3.542	4.307	2.480	2.494	3.018	1.713
	*Stachybotrys chartarum*	1.241	1.016	1.337	1.007	1.212	0.971
	*Mortierella elongata*	0.623	1.119	1.443	0.476	0.618	0.726
	*Setophoma terrestris*	0.960	0.464	0.496	1.094	1.272	0.615
	*Fusarium solani*	0.875	0.645	0.862	1.018	1.032	0.338
	*Trichoderma harzianum*	0.253	1.218	0.972	0.869	1.021	0.332
	*Preussia globosa*	0.733	0.675	1.030	0.587	0.601	0.591

**Fig. 7:**
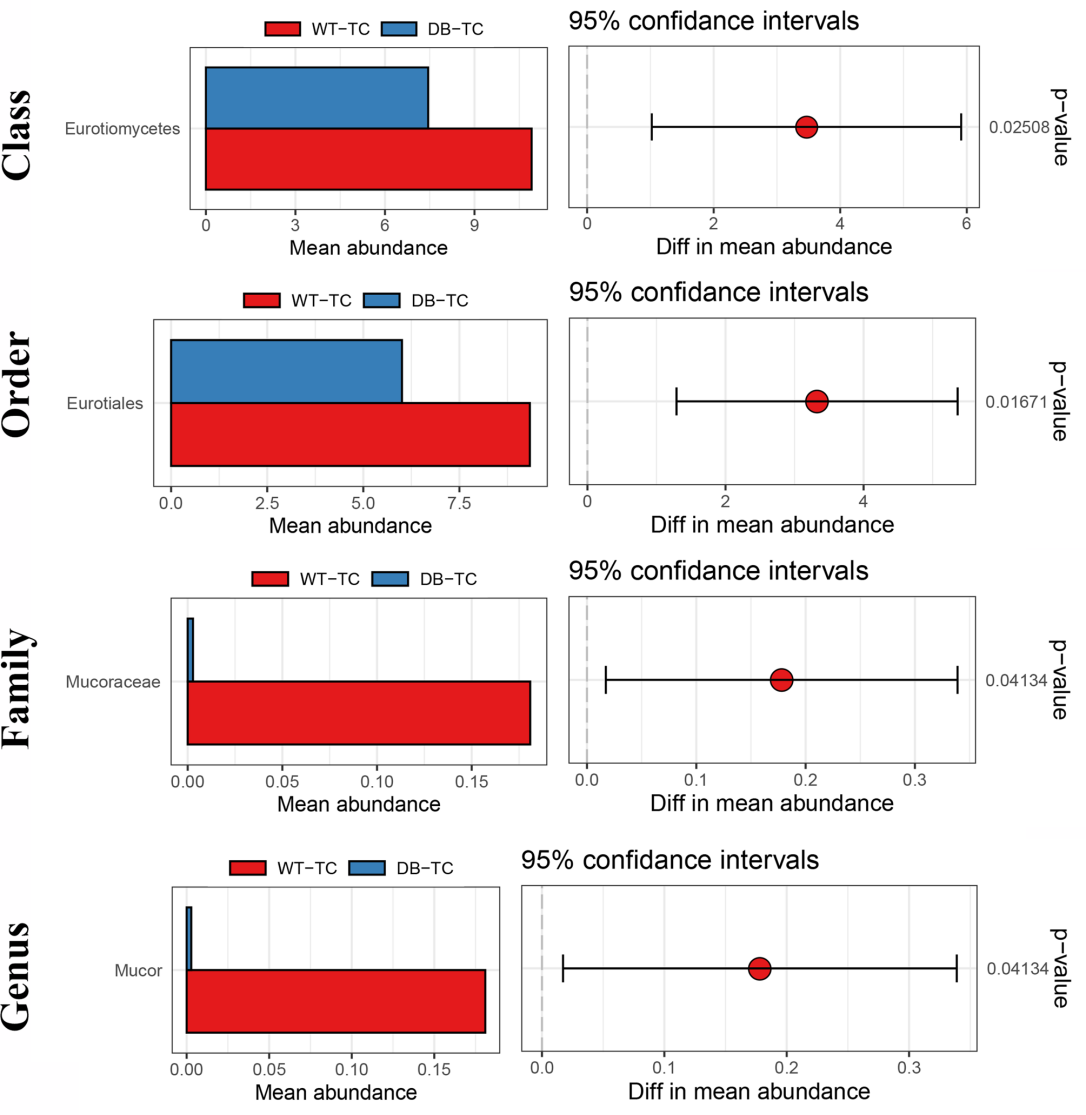
The fungal taxonomic composition and abundance differences between the WT-TC and DB-TC groups based on ITS sequencing. The taxonomic composition of the fungal community at the phylum, class, order, family, and genus levels is presented. *Ascomycota*, *Sordariomycetes*, *Sordariales*, *Bolbitiaceae*, and *Agrocybe* were the dominant taxa found in the samples. At the species level, the most high-frequency OTU1 was identified as *Agrocybe pediades*, *Serendipita indica*, *Humicola grisea*, and *Rhizopus arrhizus*. The abundance of Eurotiomycetes, Eurotiales, Mucoraceae, and Mucor was significantly reduced in the diabetic group compared to the control group (*p* < 0.05)

Furthermore, the abundance of *Eurotiomycetes*, *Eurotiales*, *Mucoraceae*, and *Mucor* was significantly lower in the diabetic group compared to the control group (Fig. 7, *p* < 0.05).

### Functional prediction of 16 S rDNA and ITS sequencing communities

**Fig. 8:**
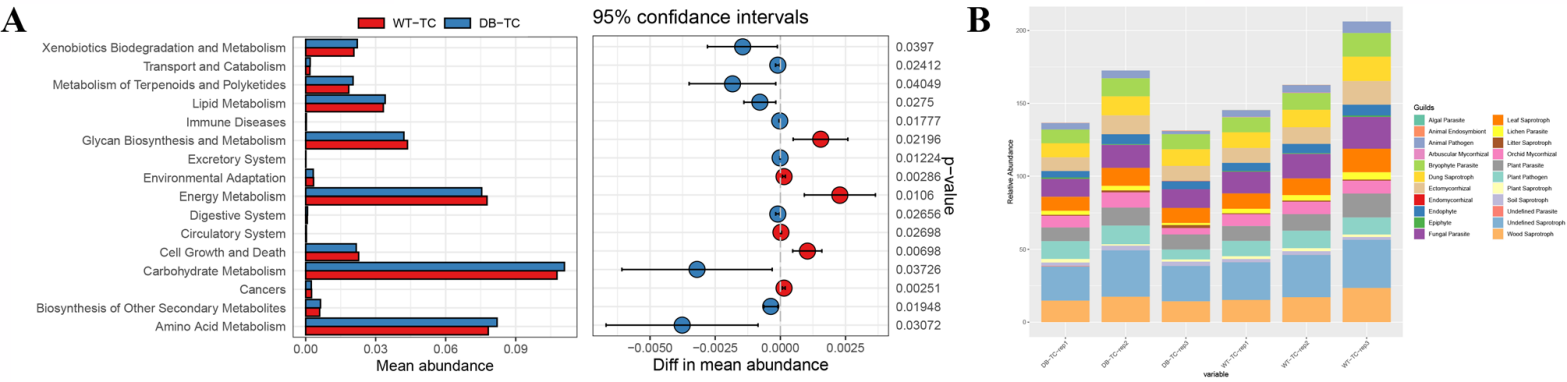
Functional prediction of 16 S rDNA and ITS sequencing communities. (A) The predicted functional analysis of the KEGG pathways for all samples using Tax4Fun software. The top 16 KEGG secondary functional pathways with relatively high abundance are displayed, including pathways related to carbohydrate, capacity, lipid, terpene and polyketides, amino acid metabolic pathways, biodegradation and metabolism, environmental adaptation, digestion, circulatory and excretory systems, cancer and immune diseases, and cellular process pathways of cell growth and death. (B) The relative abundance of 22 fungal functional taxa (excluding unassigned taxa) analyzed using the FUNGuild tool. The functional taxa are ranked by abundance, and the results indicate that “*Bryophyte Parasite-Dung Saprotroph-Ectomycorrhizal-Fungal Parasite-Leaf Saprotroph-Plant Parasite-Undefined Saprotroph-Wood Saprotroph*” had the highest abundance among all samples, followed by undefined *saprotroph* and then *orchid mycorrhizal*

Tax4Fun software was used to perform functional annotation of KEGG pathways based on the species annotation and abundance information of OTUs. The abundance information of each pathway and KO ID was counted, and 16 KEGG secondary functional pathways with relatively high abundance are shown in Fig. 8A. The predicted functional analysis revealed that all samples were related to carbohydrate, capacity, lipid, terpene and polyketides, amino acid metabolic pathways, biodegradation and metabolism, environmental adaptation, digestion, circulatory and excretory systems, cancer and immune diseases, and cellular process pathways of cell growth and death.

Additionally, a predicted functional analysis of all treated fungal communities was conducted using the FUNGuild tool. Figure 8B shows the relative abundance of 22 fungal functional taxa (excluding unassigned taxa), and the results indicated that “*Bryophyte Parasite-Dung Saprotroph-Ectomycorrhizal-Fungal Parasite-Leaf Saprotroph-Plant Parasite-Undefined Saprotroph-Wood Saprotroph*” had the highest abundance among all samples, followed by undefined *saprotroph* and then *orchid mycorrhizal*.

### Comparison of bacterial and fungal diversity

**Fig. 9:**
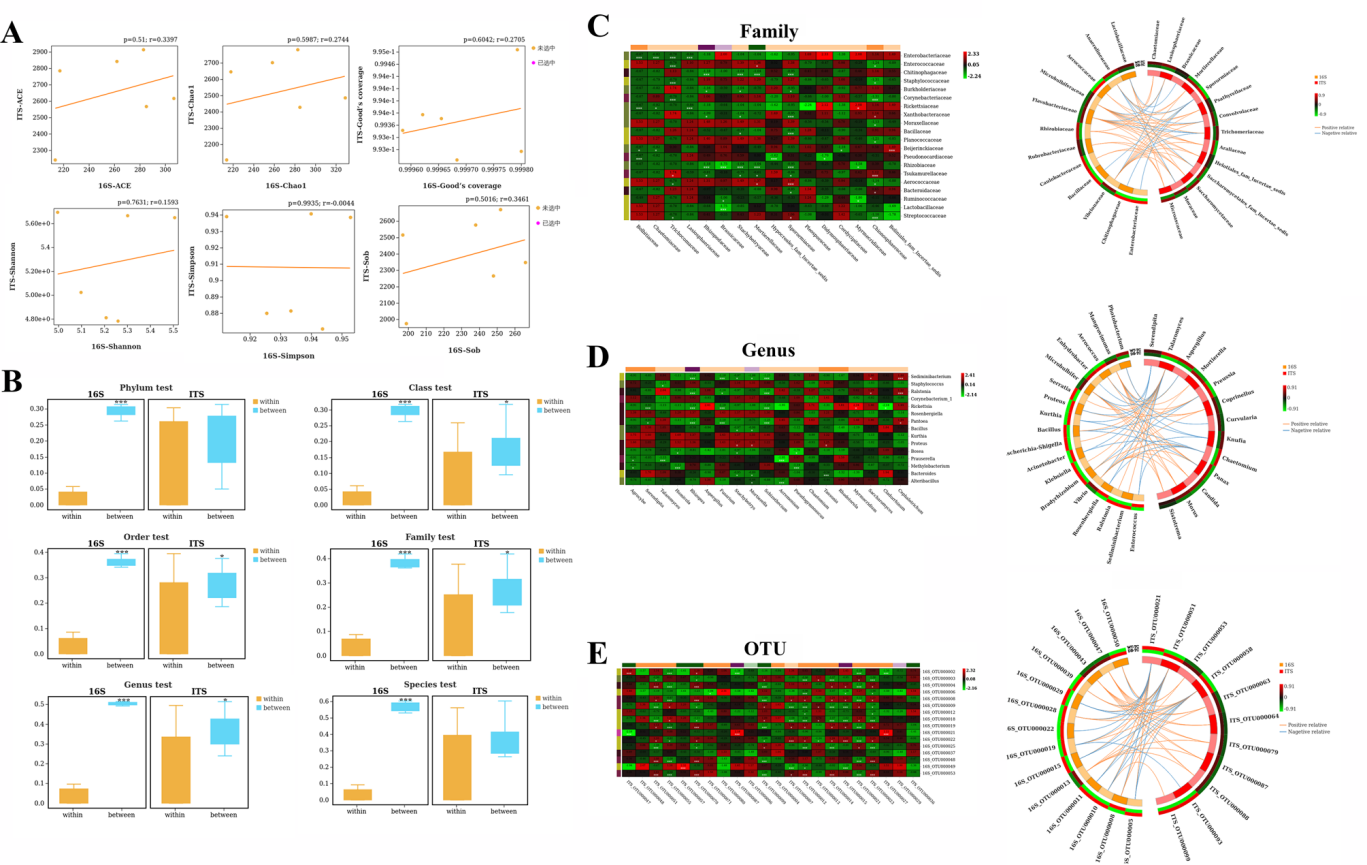
Comparison of bacterial and fungal diversity. (A) The correlation scatter plots of bacterial and fungal species diversity indices, indicating no significant correlation between the two at the alpha diversity level. (B) The statistical test results for within-group and between-group distance differences, as well as the combined distance box plot, demonstrating that grouping differences affected bacteria at the Phylum, Order, Family, Genus, and Species levels and fungi only at the Order, Family, and Genus levels. (C-E) The correlation analyses between bacteria and fungi at the family, genus, and OTU levels using the Sparse Correlations for Compositional data model. The heat map and circos plots are based on the sPLS screening of strongly correlated species, with the internal circos presenting correlation results and the external heat map displaying specific species abundance trends. The results show that there were correlations between bacteria and fungi at all three taxonomic levels, indicating interactions between bacteria and fungi

To assess the consistency of bacterial and fungal richness levels, correlation coefficients were used to analyze the trends of bacterial and fungal species in each subgroup at the species diversity (alpha diversity) level. Correlation scatter plots were then created to visualize the effect of correlation. The results indicated that there was no significant correlation between changes in bacterial and fungal species diversity, suggesting that bacteria and fungi responded differently to grouping differences (Fig. 9A). At the beta diversity correlation level, statistical tests were performed for within-group and between-group distance differences, and the combined distance box plot showed that grouping differences affected bacteria at the Phylum, Order, Family, Genus, and Species levels, while fungi were only affected at the Order, Family, and Genus levels (Fig. 9B).

The Sparse Correlations for Compositional data model was used to analyze the correlations between bacteria and fungi at different taxonomic levels (Fig. 9C and E). Species with mean values of abundance greater than 0.1% at the family, genus, and OTU levels were screened for correlation analysis. A combined heat map and circos were plotted based on the sPLS screening of strongly correlated species. The internal circos presented the correlation results, including positive and negative correlations and the intensity of the correlation. The external heat map showed the specific species abundance trends. The results showed that there were correlations between bacteria and fungi at the family, genus, and OTU levels, indicating that there were some interactions between bacteria and fungi.

## Discussion

The risk of bacterial infections in the conjunctiva, such as acute infectious conjunctivitis, is significantly higher in diabetic patients [30]. The normal flora of the conjunctiva inhibits the growth and invasion of pathogenic bacteria by limiting their nutrition and space for growth and enzyme secretion. The most frequently isolated microorganisms from the normal conjunctival flora include *Staphylococcus epidermidis*, *Staphylococcus aureus*, *Propionibacterium acnes*, *Corynebacterium*, *Streptococcus*, and *Haemophilus* [31, 32]. Orhan Ateş et al. measured conjunctival flora in type 1 and type 2 diabetic patients, as well as controls without any ocular disease, and found that *Staphylococcus epidermidis* and *Staphylococcus aureus* were present in all samples, with higher levels in type 2 diabetes [33]. Previous studies that used conventional bacterial or fungal culture methods to study normal ocular surface fungi found that the most common fungal genera were *Aspergillus*, *Rhizopus*, *Pseudostelium*, and *Penicillium*, respectively [34]. However, conventional culture methods have limitations in the study of microbial communities as certain pathogens are difficult to grow under conventional conditions, and many fungi exist in nature that cannot yet be cultured, resulting in an incomplete picture of the ocular surface fungal community [35]. Therefore, the microbial detection level of conventional culture methods is lower compared to molecular macrogenomics or 16 S rDNA or ITS sequencing [36]. In this study, we combined transcriptome and microbial diversity analysis of conjunctival tissues to systematically analyze the microbial diversity in diabetic conjunctival tissues.

Initially, we employed RNA-seq to investigate the transcriptomes of two groups of mouse conjunctival tissues and identified 449 differentially upregulated genes and 1,006 differentially downregulated genes. Through functional enrichment analysis of differentially expressed genes, we observed significant enrichment in various metabolic pathways. As metabolic abnormalities, such as dyslipidemia, hyperinsulinemia, insulin resistance, and obesity, are implicated in the pathogenesis and progression of T2DM, these findings were consistent with previous studies [37]. T2DM is a systemic inflammatory disease characterized by insulin resistance or reduced metabolic response to insulin in multiple tissues, including adipose tissue, liver, and skeletal muscle, and by reduced insulin synthesis in pancreatic β-cells [38, 39]. Moreover, immunometabolic investigations have revealed a close interrelationship between metabolic status and immune processes, whereby metabolites from the host or microbiota regulate the immune response during health and disease [40]. Therefore, to gain deeper insights into the microbial community structure in healthy and diseased states, further analyses are warranted.

Subsequently, we analyzed the bacterial community structure in both groups of samples using 16 S rDNA and found that *Proteobacteria*, *Firmicutes*, *Bacteroides*, and *Acinetobacter* were the top four phyla in relative abundance in the conjunctival microbiota of healthy mice and diabetes mellitus mice. The top four genera were *Enterococcus*, *Sediminibacterium*, *Staphylococcus*, and *Ralstonia*. These results were consistent with a previous study by Li et al., which analyzed the conjunctival microbiota composition in healthy subjects and diabetic patients using 16 S rRNA [41]. However, among the top four phyla, the abundance of *Firmicutes* was significantly increased in the diabetic group compared to the healthy group, and the proportion of *Aspergillus* and *Bacteroidetes* was significantly reduced. It has been suggested that reducing the levels of *Firmicutes* in the gut can help control T2DM and reduce insulin resistance [42]. The altered *Firmicutes*: *Bacteroidetes* ratio in obese individuals is associated with an increased risk of obesity-related DM [43]. Similarly, there are studies demonstrating significantly fewer *Proteusbacillus vulgaris* in prediabetic or diabetic patients [44].

In ITS sequence analysis, most of the fungi present in the samples belonged to *Ascomycota*, *Sordariomycetes*, *Sordariales*, *Bolbitiaceae*, and *Agrocybe*. The abundance of *Eurotiomycetes*, *Eurotiales*, *Mucoraceae*, and *Mucor* was significantly reduced in the diabetic group of mice compared to the control group. These fungi were identified for the first time in diabetic conjunctival tissue. A previous study analyzing altered ocular surface fungal flora in patients with fungal keratitis found that *Ascomycota* had the highest relative abundance in healthy and fungal keratitis patients, and the relative abundance was higher in patients with fungal keratitis than in the healthy population [45]. *Nasal-orbital mucormycosis*, which is commonly seen in immunocompromised subjects and in patients with decompensated diabetes, is an acute and fulminant fungal infection caused by vascular invasive fungi of the Mucoraceae [46].

In addition, we found that the bacterial alpha diversity index was lower in the diabetic group than in the healthy group, which is contrary to previous studies. For example, Zhu et al. found that the alpha diversity of the conjunctival flora was significantly higher in T2DM patients than in controls [47]. However, in a study by Matsha et al., the alpha diversity of oral microorganisms was found to be lower in diabetic patients compared to prediabetic or normoglycemic patients based on the Chao1 index [44]. Microbial diversity seems to be related to the duration of T2DM. For instance, the conjunctival microbiome diversity was found to be lower in T2DM patients with longer disease duration, which may indicate lower diversity dysregulation at the ocular surface later in the disease [48]. The lower conjunctival microbiome alpha diversity in T2DM patients with a disease duration greater than 15 years may also indicate a higher risk of ocular infection [47].

Furthermore, we did not find a significant difference in the fungal alpha diversity index, which is in line with the study by Bataineh et al. Predictive analyses of microbial community function revealed associations between bacterial communities and metabolic pathways, digestive, circulatory, and excretory systems, cancer and immune diseases, and cellular process pathways of cell growth and death. The mammalian immune system plays a critical role in maintaining the balance of the microbial community in vivo, thus ensuring that the reciprocity of the host-microbe relationship is maintained. At the same time, parasitic bacteria profoundly shape mammalian immunity [49].

Finally, the integration of 16 S rDNA and ITS analyses revealed no significant correlation between changes in bacterial and fungal species diversity, indicating that bacteria and fungi respond differently to grouping differences. We found that bacteria and fungi are related at three taxonomic levels—family, genus, and OTU—suggesting some interactions between bacteria and fungi. Other studies have reported the effect of fungi on bacterial community composition [50, 51]. Fungi may be key components of the microbial community and have important effects on the gut ecosystem and possibly on host health [52]. However, the potential role of fungi and their interactions with the host, other members of the gut flora, and metabolic health require further understanding. We also found multiple fungal species colonizing the conjunctiva, yet no mice developed fungal infections, suggesting that there may be some interaction between bacteria, fungi, and host immunity in the maintenance of ocular surface health, which also needs further investigation.

## Data Availability

The datasets used and/or analyzed during the current study are available from the corresponding author upon reasonable request. **Competing interests**: The authors declare that they have no competing interests.
